# Is cardiomyopathy intrinsic to Marfan syndrome?

**DOI:** 10.1038/s41390-025-03899-0

**Published:** 2025-02-03

**Authors:** Alexis M. Wolf, Carolyn M. Wilhelm, James Strainic, Timothy J. Mead

**Affiliations:** 1 Division of Pediatric Cardiology, University Hospitals Rainbow Babies & Children’s Hospital, Cleveland, OH USA; 2 Department of Pediatrics, Case Western Reserve University School of Medicine, Cleveland, OH USA

Marfan syndrome (MFS) is an autosomal dominantly inherited connective tissue disorder caused by fibrillin-1 (FBN1) gene mutations with a prevalence of 6.5/100,000.^1^ There are more than 3000 different FBN1 pathogenic variants with difficult-to-predict genotype-phenotype correlations. These mutations are attributed to classical findings of aortic dissection and valvular disease, including mitral valve prolapse, which are hallmarks of MFS. However, their roles in cardiomyopathy are well less understood. FBN1 is thought to play a role in sustaining proper cardiac function by contributing to the diastolic and systolic properties of the myocardium. Alterations to FBN1 cause abnormal mechano-signaling of the microfibrils which may cause inadequate compensation with reduced elastic recoil, myocardial stretch, and impaired muscle contractility in the presence of volume or pressure overload.^2^ A recent human in vitro study demonstrated that dysfunctional cardiac myocytes and fibroblasts lead to abnormal early development of cardiac myocytes and play a role in intrinsic MFS cardiomyopathy.^3^

To date, multiple studies in the adult population demonstrate intrinsic cardiomyopathy in MFS with left ventricular (LV) dilation and dysfunction. Cardiac magnetic resonance imaging (CMR) revealed 21.7% (*n* = 69) of cohort patients have diminished cardiac function and left ventricular ejection fraction (LVEF) less than 55% without significant aortic or mitral valve regurgitation and no history of arrhythmias or prior valve surgery. The ratio of peak early diastolic to peak systolic strain rate was significantly reduced in MFS patients compared to healthy controls, indicating evidence of diastolic dysfunction (*p* < 0.0001).^4^ A second study demonstrated biventricular dysfunction via CMR with right ventricular ejection fraction (RVEF) and LVEF significantly reduced in MFS patients (*p* < 0.005).^5^ A further study utilized 3-dementional speckle tracking echocardiography (3D-STE) and found that compared to controls, MFS patients had significantly lower 3D-STE LVEF (*p* = 0.0001), LV global longitudinal strain (*p* = 0.0001), LV global circumferential strain (*p* = 0.0001) and LV global area strain (*p* = 0.0001); no significant difference was found using conventional echocardiography when evaluating EF, however M-mode derived MAPSE (*p* = 0.005) and Doppler derived aortic velocity time integral (*p* = 0.001) were significantly lower compared to controls.^6^ The role of FBN1 mutations that are causative in pediatric cases of cardiomyopathy, however, are less understood.

In this issue of Pediatric Research, KneuBel et al. identified this gap in knowledge regarding risk factors for pediatric MFS cardiomyopathy and sought to determine the prevalence cardiomyopathy characteristics: low cardiac index (CI) for age, LVEF < 50%, or presence of diastolic dysfunction.^7^ Including eighty-three pediatric MFS patients at the University Heart and Vascular Center UKE Hamburg from 2014 to 2020, this single-center retrospective observational study aims to identify predictors of MFS cardiomyopathy and establish a scoring system to quantify occurrence risk in this patient group. The authors identified specific genetic variants in the FBN1 gene associated with an increased risk of developing cardiomyopathy. Genetic variants were classified as missense variants, splice site variants, or variants introducing a premature stop codon. The major finding of the study was the significant presence of cardiomyopathy characteristics (CMCs) in children and adolescents with MFS, with low rates of heart failure symptoms.

The median age of participants was 12.5 years, ranging 0.4 to 22.3 years. Marfan cardiomyopathy, as defined by the study group, was identified in 33 (39.8%) patients. Low CI was observed in 78.8% (*n* = 26), reduced LVEF in 6.0% (*n* = 5), and evidence of diastolic dysfunction was present in 11.3% (*n* = 8) of the study population. Importantly, the study found that only 4.8% (*n* = 4) of patients had symptoms of heart failure according to the NYHA classification, and all patients under 6 years old had no heart failure symptoms based on Ross classification. The analysis identified four factors that independently predicted the presence of cardiomyopathic characteristics: systemic score ≥ 7 based on revised Ghent criteria (RGC), a variant in FBN1 introducing a premature stop codon, a splice site variant, and a missense variant involving cysteine. These findings suggest that cardiomyopathy may be present long before overt symptoms, and it can be predicted that any MFS patients with more severe features will likely develop intrinsic cardiomyopathy in the future.

While the results of this study are promising, there are some limitations acknowledged by the authors. First, this is a retrospective single center study with a small sample size. It is unknown if patients with the defined CMCs will develop cardiomyopathy in the future. Additionally, while patients were included who had known MFS by the RGC, there were patients included with a likely pathogenic variant in the FBN1 gene, that may not have a diagnosis of MFS. It should also be noted that there were patients included who had moderate mitral regurgitation, and surgical aortic valve replacement before study inclusion, which could confound the results. It is understandable that the pediatric population sample size is minimal compared to the adult population. It also must be noted that the reference for CI used in this study was from a Chinese cohort, which may not be generalizable to the rest of the population as there is intrinsic variability in height and weight. Finally, the study does not define what constitutes diastolic dysfunction in the 8 patients.

Regarding their scoring system, the statement that “a cardiomyopathy score of 1 or less was associated with a 27.4% estimated probability of Marfan cardiomyopathy” could better be clarified. Furthermore, the Teichholz method is no longer recommended for clinical use, as volume measurements based on linear measurements are heavily reliant on geometrical assumptions of the LV shape. Three-dimensional echocardiography is now considered the standard method to measure LVEF by echocardiography, as it doesn’t require these geometric assumptions.^8^

Despite the limitations of the study, the concepts and data are promising. In a study by Cook et al., it was demonstrated in a mouse model of MFS that reduced fibrillin-1 production by cardiomyocytes is sufficient to precipitate dilative cardiomyopathy in cardiomyocytes.^9^ Furthermore, deficient fibrillin-1 leads to altered myocardial transforming growth factor-β (TGF-β), which can lead to myocardial structural changes due to its association with fibrosis in pressure loaded heart failure.^9^ While MFS cardiomyopathy has been mainly shown in the adult population, recent studies have described it in the pediatric population. In Weigand et al., CMR showed that among patients with absent/mild LV volume load, indexed LV end-diastolic volume (EDV) was significantly increased above normative values and LVEF was abnormal in 48% of pediatric MFS patients, suggesting intrinsic cardiomyopathy.^10^ Furthermore, Connell et al. demonstrated using CMR that 48% of their MFS cohort had an LVEF < 55%, worse global and longitudinal circumferential strain, and were associated with diminished LVEF, but without associated aortic dilation.^1^ These studies are promising and additive to the current study, but with small sample size and retrospective designs.

The KneuBel et al. study makes the case for assigning clinical predictors of primary cardiomyopathy in MFS, which would be of benefit for management of their future operative and post-operative courses.^7^ Xu et al. demonstrated that MFS patients had a lower postoperative LVEF after a Bentall procedure compared to non-MFS matched controls, and MFS was an independent risk factor for adverse postoperative cardiogenic events during patients’ hospital stay, implying intrinsic cardiomyopathy.^11,12^ In addition, it has been shown that arrhythmia is a recognized relevant manifestation in MFS in the adult population, which could help predict risk of sudden cardiac death. MFS patients were shown to have a higher median of premature atrial and ventricular contractions than matched controls. Ventricular ectopy was associated with LV size, mitral valve prolapse, and repolarization abnormalities. This suggests that intrinsic MFS cardiomyopathy may be a proarrhythmogenic substrate similar to what is seen in other cardiomyopathies that allow perpetuation of severe ventricular arrhythmias.^2^ The current KneuBel study can be built upon and improved with implementation of prospective multi-center studies in order to increase power and representation of the general pediatric population, defining parameters of diastolic dysfunction, and utilizing the ideal methods of measuring LV systolic function with CMR and 3D-STE.^7^ The study’s key findings include that a significant proportion of the pediatric patients with MFS exhibited signs of cardiomyopathy despite being asymptomatic and that FBN1 mutations with a missense variant in cysteine had the highest risk for MFS cardiomyopathy. This key observation suggests that other than assessing aortic or valvular abnormalities, which is already routine for monitoring patients with MFS, it would benefit patients to also monitor for intrinsic cardiomyopathy as part of the standard cardiac surveillance in MFS.
